# Impact of morphological variations on preoperative and postoperative central corneal thickness in congenital cataract: a retrospective observational study

**DOI:** 10.1007/s10792-025-03671-7

**Published:** 2025-07-09

**Authors:** Suzu Deie, Yoshiaki Kiuchi, Kaori Komatsu, Kazuyuki Hirooka, Hirokazu Sakaguchi

**Affiliations:** Department of Ophthalmology and Visual Science, Graduate School of Biomedical Sciences, Hiroshima University, Hiroshima, 734-8551 Japan

**Keywords:** Cataract, Central corneal thickness, Glaucoma, Secondary glaucoma, Pediatric, Intraocular disease

## Abstract

**Purpose:**

Central corneal thickness (CCT) has been reported to be thicker in aphakic and pseudophakic eyes after congenital cataract surgery. We aimed to investigate the factors influencing preoperative CCT and changes in CCT after cataract surgery.

**Methods:**

We included 62 eyes of 39 patients with congenital cataracts who underwent cataract surgery at Hiroshima University between February 2006 and July 2022. Pre- and postoperative CCT were measured using a noncontact specular microscope or ultrasound pachymeter. Cataracts were categorized into five morphologies: anterior, posterior, nuclear, total, and others (aculeiform, calcific, cortical, lamellar, pulverulent, and membranous). Statistical analyses were conducted using JMP software, with significance set at *P* < 0.05.

**Results:**

No significant differences in CCT were observed between bilateral and unilateral cataracts or between operated and fellow eyes with unilateral cataracts. However, when cataracts were categorized according to morphology into five categories, namely, anterior cataract, posterior cataract, nuclear cataract, total cataract, and others, the others group exhibited significantly thicker CCT than did the control group (566.6 ± 76.2 µm vs. 525.9 ± 31.2 µm, *P* = 0.032). Preoperative CCT decreased inversely with age. Postoperative CCT increased significantly compared with preoperative measurements (544.0 ± 35.9 µm vs. 534.5 ± 28.4 µm, *P* = 0.021).

**Conclusion:**

This study highlights that congenital cataract of specific morphologies, particularly those classified in the others group, affect CCT. Preoperative CCT decreased with age. The increase in postoperative CCT suggests that surgical intervention can affect corneal thickness.

## Introduction

Congenital cataract is a rare ocular disease with prevalence rates varying from 0.6 to 9.3 per 10,000 live births [1]. However, it is a significant cause of childhood visual impairment, representing approximately 5–20% of childhood blindness worldwide [2]. Early diagnosis and treatment are crucial to prevent the development of amblyopia and other vision-related conditions. The primary treatment for congenital cataracts is surgical removal, often followed by intraocular lens implantation. However, surgical interventions can lead to various postoperative complications. Glaucoma is one of the most common complications of congenital cataract surgery, with an incidence of 6.1–26% [3–8]. Central corneal thickness (CCT) is a critical parameter as it affects the accuracy of intraocular pressure measurements; it plays an essential role in diagnosing and managing glaucoma, especially in children with limited testing capabilities, including that of visual field testing [9]. Understanding the factors influencing CCT in congenital cataracts is essential for the optimization of surgical outcomes and postoperative care. Previous studies have reported that CCT is thicker in aphakic and pseudophakic eyes after congenital cataract surgery [10–12]. The relationship between cataract morphology and CCT has not been extensively studied, and reports have been limited [13, 14]. If specific cataract morphologies are associated with preoperative CCT variations, these should be considered during surgical planning and postoperative management. This study aimed to investigate the factors influencing preoperative CCT in eyes with congenital cataracts and evaluate changes in CCT following cataract surgery. The results will help provide a comprehensive understanding of CCT variations in congenital cataracts by comparing CCT measurements between different groups, including those of bilateral and unilateral cataracts, and by analyzing the influence of cataract morphology.

## Materials and methods

### Study design and population

The University of Hiroshima Institutional Review Board approved this retrospective study (approval no. E-2113). Following the principles outlined in the Declaration of Helsinki, written surgical consent for cataract extraction was obtained from the parents of patients. Consent for inclusion in the current study was waived because corneal thickness measurement is a routine part of cataract evaluation. For the control group, consent was waived because the patients were scheduled for strabismus surgery and corneal evaluation was required prior to surgery.

### Objectives

The primary objective was to compare preoperative CCT across cataract morphologies, age, and laterality and, in an exploratory multivariable model, to identify independent factors associated with preoperative CCT. The secondary objectives were to quantify the postoperative change in CCT and to explore whether this change differed across cataract morphologies.

### Data collection and study groups

Patients aged ≤ 12 years who were diagnosed with congenital cataract and underwent cataract surgery at Hiroshima University between February 2006 and July 2022 were eligible for inclusion in the study. Patients with traumatic cataracts or coexisting ocular conditions that could affect CCT, such as glaucoma, corneal trauma, previous intraocular surgery, Down syndrome, and anterior segment malformations other than cataracts (e.g., aniridia), and those with incomplete data, were excluded. In the control group, 31 patients aged between 48 and 96 months with no ocular diseases, except strabismus or amblyopia, were evaluated.

Data on age, sex, laterality, cataract morphology, and pre- and post-operative CCT findings were collected from medical records. Preoperative CCT measurements were performed using a noncontact specular microscope (Tomey EM-3000; Tomey Corporation, Nagoya, Japan) in cooperative children (n = 53 eyes). If a patient could not follow the instructions, or if measurements with a specular microscope were impossible, CCT measurements were conducted using an ultrasound pachymeter (Tomey SP-100; Tomey Corporation, Nagoya, Japan) prior to cataract extraction under general anesthesia (n = 9 eyes). Corneal thickness was measured three times at the center of each cornea, and the mean of the three measurements was used. All postoperative measurements (n = 20 eyes) were performed using a noncontact specular microscope. For postoperative CCT analysis, only eyes in which reliable measurements could be obtained during follow-up visits were included; uncooperative patients, particularly younger children, were excluded. Cataracts were categorized, according to morphology, into five categories suggested by Thau et al. [13]: (1) anterior cataract, including anterior polar, anterior lenticonus, anterior cortical, and anterior capsular and subcapsular cataracts; (2) posterior cataract, including posterior polar, posterior lenticonus, posterior cortical, and posterior subcapsular cataracts; (3) nuclear cataract, including opaque embryonic or fetal nuclear cataracts; (4) total cataract; and (5) others, including aculeiform, calcific, cortical, lamellar, pulverulent, and membranous cataracts.

### Statistical analysis

All statistical analyses were conducted using JMP Pro software version 16 (SAS Inc., Cary, NC, USA). Quantitative data are expressed as mean ± standard deviation and range. Clinical characteristics were compared among the control, bilateral cataract, and unilateral cataract groups using analysis of variance for continuous variables and the chi-square test for categorical variables. Dunnett’s test was used for comparisons of corneal thickness values with controls, and a paired *t*-test was used for comparisons with the same patients. A multiple linear regression analysis was performed with preoperative CCT as the dependent variable and age at surgery, sex, laterality (bilateral vs. unilateral), and cataract morphology as independent variables. In a multiple linear regression analysis, morphology was dummy coded with posterior cataract as the reference category; therefore, coefficients represented the adjusted difference in CCT relative to posterior morphology. We quantified inter-eye correlation (ICC = 0.90), as both the eyes of some patients were included in this study. A mixed-effects model with patient ID as a random intercept showed near-zero random variance (Wald *P* = 0.39). Therefore, coefficients were reported from the ordinary least-squares model. A *P*-value < 0.05 was considered indicative of statistical significance.

## Results

A total of 62 eyes from 39 patients (21 females and 18 males) with congenital cataracts were included in this study. The control group included 62 eyes from 31 patients (17 females, 14 males). The patient characteristics are summarized in Table 1. The mean age for cataract surgery was 62.5 ± 44.5 (0–152) months. Twenty-three patients had bilateral cataracts, and 16 had unilateral cataracts. Of the 16 patients with unilateral cataracts, 8 had cataracts in the right eye, and 8 had cataracts in the left eye. The control group had a mean age of 73.5 ± 14.0 (51–96) months. There were no significant differences in age, sex, laterality, or CCT between the groups. During the follow-up period, one patient with unilateral cataract developed glaucoma in the same eye, requiring surgical intervention.

**Table 1:** Clinical characteristics of patients with cataracts and the controls

	Controls	Patients with cataracts	*P*-value
Number of eyes/individuals	62/31	62/39	–
Age in months^a^	73.5 ± 14.0 (51–96)	62.5 ± 44.5 (0–152)	0.19
Sex (male:female)	14:17	18:21	0.93
Laterality (right:left)	31:31	31:31	1.00
CCT^a^ (µm)	525.9 ± 31.2	539.7 ± 63.7	0.13

^a^Data are expressed as mean ± standard deviation (range)

CCT, Central corneal thickness

**Fig. 1:**
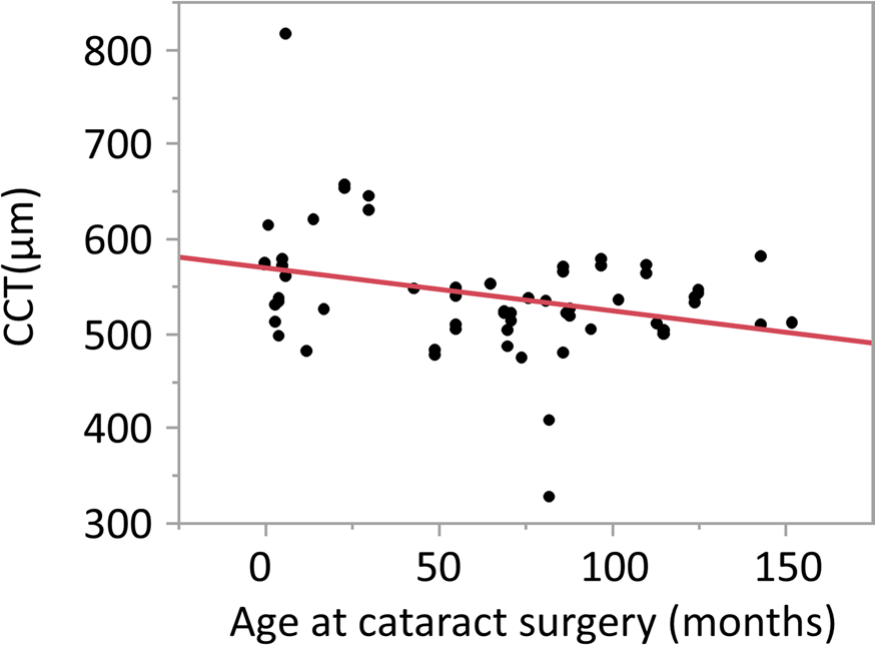
Relationship between age (months) at cataract surgery and central corneal thickness (CCT). CCT and age at cataract surgery showed a negative correlation (*r* = − 0.309, R^2^ = 0.095, *P* = 0.015)

Figure 1 shows scatter diagrams between preoperative CCT and age in patients with cataracts. There was a negative correlation (*r* = − 0.309, *P* = 0.015). When comparing preoperative CCT measurements in the operated and contralateral eyes in the unilateral cataract group, the mean CCT was 548.8 ± 77.4 µm (range, 474–816 µm) in the operated eyes and 545.4 ± 84.1 µm (range, 466–843 µm) in the fellow eyes. No significant differences were observed (*P* = 0.57, paired *t*-test). Table 2 compares the preoperative CCT measurements between the control and bilateral cataract groups and between the control and unilateral cataract groups. The mean CCT was 525.9 ± 31.2 µm in the control group, 536.9 ± 56.1 µm in bilateral cataracts, and 548.8 ± 77.4 µm in unilateral cataracts in the operated eyes. Compared with the controls, no significant differences were observed in bilateral (*P* = 0.65) or unilateral cataracts (*P* = 0.35), using Dunnett’s test. Table 3 shows a comparison of preoperative CCT measurements based on cataract morphology. Cataract morphology analysis revealed anterior cataract in 9 eyes, posterior cataract in 25, nuclear cataract in 6, total cataract in 7, other morphologies in 13, and unknown in 1. Compared with the control group, significant differences were observed only in the others group, using Dunnett’s test. In the others group, 7 eyes had lamellar cataracts, 4 had cortical cataracts, and 2 had pulverulent cataracts. Mean CCT in lamellar cataracts was 542.4 ± 27.0 µm, in cortical cataracts was 609.0 ± 121.4 µm, and in pulverulent cataracts was 567.5 ± 2.5 µm. However, because of the small sample size, statistical comparisons between these subtypes were not performed. To adjust for potential confounding, a multiple linear regression analysis was performed with preoperative CCT as the dependent variable and age at surgery, sex, laterality, and cataract morphology as covariates (Table 4). Age remained an independent predictor, with CCT thinning by 0.45 µm for every additional month of age (*P* = 0.025). After adjustment, the eyes classified in the others morphology group showed a tendency toward thicker corneas (+ 26 µm relative to posterior cataract); however, this finding was not statistically significant (*P* = 0.108). Sex and laterality again showed no association with CCT (both *P* > 0.25).

**Table 2:** Comparison of preoperative central corneal thickness

	N (eyes)	Mean (µm)	SD (µm)	Range (µm)	*P*-value
Control group	62	525.9	31.2	436–603	–
Bilateral cataracts	46	536.9	56.1	327–657	0.65
Operated eye with unilateral cataract	16	548.8	77.4	474–816	0.35

The *P*-value was tested with Dunnett’s test

SD, Standard deviation

**Table 3:** Comparison of central corneal thickness by cataract morphology

	N (eyes)	Mean (µm)	SD (µm)	Range (µm)	*P*-value
Control group	62	525.9	31.2	436–603	–
Cataract group					
Anterior cataract	9	521.3	30.5	486–578	1.00
Posterior cataract	25	524.0	58.0	327–645	1.00
Nuclear cataract	6	538.0	4.86	532–546	0.98
Total cataract	7	562.3	77.9	408–657	0.26
Others	14	566.6	76.2	513–816	0.03*

The *P-*value was tested with Dunnett’s test

SD, Standard deviation

*P < 0.05

**Table 4:** Multivariable linear regression analysis for preoperative central corneal thickness

Predictor	β (µm)	SE	95% CI	*P*-value
Age at cataract surgery	− 0.453	0.193	− 0.85 to − 0.06	0.025*
Sex (female vs. male)	− 7.98	9.19	− 26.4 to 10.4	0.389
Laterality (unilateral vs. bilateral)	− 8.96	10.4	− 29.9 to 12.0	0.395
Morphology				
Anterior–Posterior	− 29.6	19.0	− 67.8 to 8.58	0.126
Nuclear–Posterior	9.21	22.1	− 35.1 to 53.6	0.679
Total–Posterior	21.3	20.6	− 20.0 to 62.6	0.305
Others–Posterior	25.9	15.9	− 5.92 to 57.8	0.108

Values are two-sided t-tests from ordinary least-squares regression. Posterior cataract serves as the reference category (β = 0)

SE, Standard error; CI, Confidence interval

*P < 0.05

**Fig. 2:**
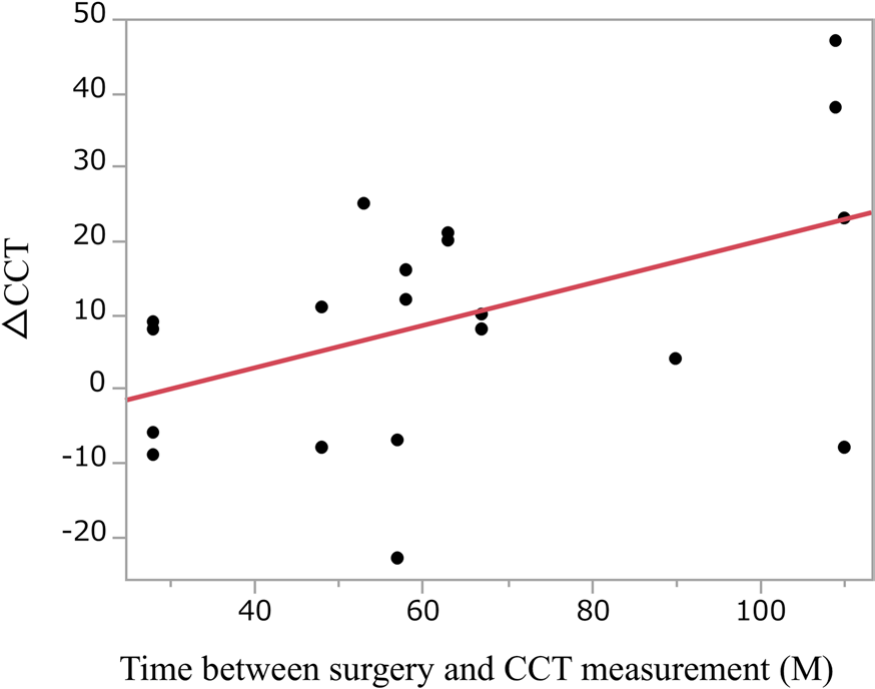
Postoperative change in central corneal thickness (CCT) as a function of time since surgery. CCT increased by 0.29 µm per month after surgery (β = 0.29, R^2^ = 0.22 [*r* = 0.47], *P* = 0.035)

Of the 62 eyes, 20 with cataracts underwent both pre- and post-operative CCT measurements. The mean time between cataract surgery and measurement of postoperative CCT was 64.0 ± 27.4 months (range, 28–110 months). The mean CCT significantly increased from 534.5 ± 28.4 (range, 486–578) µm preoperatively to 544.0 ± 35.9 (range, 495–618) µm postoperatively (*P* = 0.021, paired *t*-test, Table 5). A simple linear regression showed that CCT increased by 0.29 µm per month after surgery (β = 0.29, *P* = 0.035, R^2^ = 0.22, Fig. 2). Thus, approximately 22% of the variance in the increase of CCT was explained by the postoperative interval. Among the 20 eyes with paired data obtained by the same instrument (specular microscope at both time-points), mean CCT increased from 530.4 ± 27.8 µm preoperatively to 538.8 ± 30.6 µm postoperatively (n = 17, paired t-test, *P* = 0.018). When stratified by cataract morphology, a significant increase was observed only in the others group (*P* = 0.023, Table 5). No significant change was detected in anterior or posterior cataracts, and no nuclear cataracts were available for analysis. The total cataracts subgroup was too small for statistical analysis. For the corneal endothelial cell measurement of CCT, 14 of the 20 eyes underwent evaluation of corneal endothelial cell density (CECD). A significant difference was observed between the mean preoperative and postoperative CECD (3447 ± 288 cells/mm^2^ vs. 3193 ± 283 cells/mm^2^, *P* < 0.01, paired *t*-test).

**Table 5:** Pre- and postoperative central corneal thickness by cataract morphology (n = 20 eyes)

Morphology type	N (eyes)	Preoperative CCT Mean ± SD (µm)	Postoperative CCT Mean ± SD (µm)	Mean difference (µm)	*P*-value
Anterior	6	530.0 ± 34.1	539.7 ± 54.8	+ 9.7	0.43
Posterior	6	525.0 ± 20.0	530.2 ± 13.2	+ 5.2	0.35
Nuclear	0	NA	NA	NA	NA
Total	1	572.0	595.0	+ 23	NA
Others	7	541.0 ± 25.2	552.3 ± 18.5	+ 11.3	0.023*
All cases	20	534.5 ± 28.4	544.0 ± 35.9	+ 9.5	0.021*

The *P*-value was tested with paired t-tests

CCT, Central corneal thickness; SD, Standard deviation; NA, Not applicable

*P < 0.05

## Discussion

This study investigated preoperative CCT in eyes diagnosed with congenital cataracts and compared it with eyes without intraocular disease (control group). The findings indicated no significant differences in CCT between the operated and fellow eyes in unilateral cataracts or between bilateral and unilateral cataracts. However, when cataracts were categorized according to morphology, the others group showed significantly thicker CCT than did the control group. Additionally, postoperative CCT measurements were higher than preoperative measurements. The lack of significant differences in CCT between bilateral and unilateral cataracts suggests that the presence of a cataract in one or both eyes does not inherently influence corneal thickness. This finding aligns with the results of previous studies [13, 14] that have shown similar CCT measurements in various congenital cataract presentations, indicating that cataract presence alone may not be a determining factor for CCT changes. Similarly, the absence of significant CCT differences between the operated and fellow eyes with unilateral cataracts indicates that unilateral congenital cataracts do not cause specific changes in corneal thickness.

Notably, preoperative CCT was significantly higher in the others group when comparing different cataract morphologies in this study, raising questions about the underlying causes. Both the crystalline lens and cornea follow a similar course of development during embryogenesis; the epithelial cells of the cornea and crystalline lens are derived from the epidermal ectoderm. The lens vesicle separates from the surface ectoderm during the fifth week of gestation, the primitive corneal epithelium develops by the sixth week, and the migration of the neural crest cell-derived mesenchyme forms the corneal endothelium and stroma in the seventh week [15]. Lens opacity develops after the separation of the lens vesicle. While cataracts with anterior, posterior, and nuclear morphology result from some etiological effect on the lens during its development, this factor may not have a great effect on the development of the cornea. In this study, the others group, which included lamellar, cortical, and pulverulent cataracts, had greater CCT than did the control group. We suspect that these other cataracts had some effects on both the cornea and crystalline lens during the lens vesicle separation. Given the limited sample size within each subtype of the others category, we were unable to determine whether a particular morphology drove the overall increase in CCT; larger studies are required to clarify this point. The CCT in children has been reported to gradually increase with age, reaching adult levels by 5–6 years of age [16]. However, our study revealed that the CCT in pediatric patients with congenital cataracts was inversely proportional to age. This finding suggests that the corneas of children with congenital cataracts may not undergo normal developmental processes. In other words, although the precise reasons and mechanisms remain unclear, it may be suggested that the development of cataracts may influence corneal development. Considering that congenital cataracts are associated with complications such as glaucoma, it can be hypothesized that congenital cataracts affect the development of the anterior chamber angle and, by extension, the overall development of the eye. Additionally, when compared to the control group, the cataract groups tended to have thicker corneas, which is likely due to the younger age of the patients (Table 1). However, in a subset of cataract eyes within the same age range as the control group (51–96 months, n = 24), the mean CCT was 509 ± 50.2 (range, 327–570) µm, showing a tendency to be thinner compared to the control group (*P* = 0.070, *t*-test). Further research is necessary to elucidate the exact mechanisms underlying these observations. Although the others morphology group showed a significantly thicker cornea than the control group on univariate analysis, the multivariable model revealed only a non-significant tendency toward increased thickness after adjustment for age and other covariates (+ 26 µm, *P* = 0.108). This attenuation suggests that part of the unadjusted difference may be attributable to the younger age distribution of this subgroup. Nevertheless, the persistent upward trend keeps open the possibility that specific others-type variants influence corneal development. The clinical relevance of such a difference remains uncertain, and larger, age-matched studies are required to clarify whether any specific subtype within the others category exerts a meaningful effect on corneal thickness.

As in our study, previous reports have consistently found increased postoperative CCT compared with preoperative measurements [10–12]. In our study, the mean postoperative CCT increase was 9.5 µm overall (Table 5); when stratified by cataract morphology, a significant change was detected only in the others group, whereas anterior and posterior types showed a tendency toward increase that was not statistically significant. Given that postoperative CCT was measured, on average, 64 months after surgery, part of this thickening may reflect normal age-related corneal growth rather than a purely surgical effect. However, a prospective cohort study with a larger sample size has confirmed this trend [17], further supporting the generalizability of our findings. Possible explanations for this increase include surgical trauma, postoperative inflammation, and changes in endothelial cell function, but the etiology is still unclear [18–20]. Although we found that CECD reduced after cataract surgery, this is considered to be within the range of age-related decrease in CECD [21, 22], and an increase in corneal thickness due to changes in endothelial cell function can be ruled out. The surgical intervention itself, including the implantation of intraocular lenses and the potential for corneal edema, likely contributed to the observed thickening [23]. Monitoring CCT postoperatively is critical for managing potential complications, such as elevated intraocular pressure, and for achieving visual outcomes.

Our study has some limitations. First, the retrospective nature of this study might have introduced selection bias, and the sample size, although sufficient for initial observations, may limit the generalizability of the findings. Second, the use of different instruments (specular microscope and ultrasound pachymeter) for CCT measurements, depending on patient cooperation, might have introduced measurement variability. A specular microscope and an ultrasound pachymeter were used to measure the distance between the anterior and posterior corneal surfaces. Unlike the optical reflex used by the specular microscope, ultrasound pachymetry relies on an acoustic reflection, and the exact posterior reflex point may lie between Descemet’s membrane and the anterior chamber [24, 25]. Some variability in the measurements was observed due to body movement in children [26], but this was considered to be within an acceptable margin of error. Additionally, while excluding patients with coexisting ocular conditions is necessary to isolate the effects of congenital cataracts, it may not allow the application of the findings to a broader patient population with multiple comorbidities. Future studies should include larger cohorts and prospective designs to confirm these findings and explore the mechanisms underlying the observed CCT variations. In addition, the relationship between corneal thickness and corneal diameter should be investigated as it could provide additional insights into the structural characteristics of the cornea in congenital cataracts.

In conclusion, this study showed that congenital cataracts of specific types such as lamellar, cortical, and pulverulent, affect CCT. Notably, preoperative CCT decreased with age. Additionally, the postoperative increase in CCT was not correlated with a decrease in CECD. These findings could help comprehensively understand congenital cataracts. Continued research in this area is essential to further elucidate the factors affecting CCT.

## Data Availability

Restrictions apply to the availability of these data, and they are, therefore, not publicly available. The data underlying this study are available upon reasonable request from the corresponding author (S.D, syoshito@hiroshima-u.ac.jp) with the permission of Hiroshima University Hospital.
